# Body size but not warning signal luminance influences predation risk in recently metamorphosed poison frogs

**DOI:** 10.1002/ece3.1731

**Published:** 2015-10-05

**Authors:** Eric E. Flores, Martin Stevens, Allen J. Moore, Hannah M. Rowland, Jonathan D. Blount

**Affiliations:** ^1^ Centre for Ecology and ConservationCollege of Life & Environmental SciencesUniversity of ExeterPenrynUK; ^2^ Sistema Nacional de Investigacion de Panama (SNI)Panama; ^3^ Department of GeneticsUniversity of Georgia30602AthensGeorgia; ^4^ Department of ZoologyUniversity of CambridgeCambridgeUK

**Keywords:** Aposematism, artificial models, clay models, *Dendrobates auratus*

## Abstract

During early development, many aposematic species have bright and conspicuous warning appearance, but have yet to acquire chemical defenses, a phenotypic state which presumably makes them vulnerable to predation. Body size and signal luminance in particular are known to be sensitive to variation in early nutrition. However, the relative importance of these traits as determinants of predation risk in juveniles is not known. To address this question, we utilized computer‐assisted design (CAD) and information on putative predator visual sensitivities to produce artificial models of postmetamorphic froglets that varied in terms of body size and signal luminance. We then deployed the artificial models in the field and measured rates of attack by birds and unknown predators. Our results indicate that body size was a significant predictor of artificial prey survival. Rates of attack by bird predators were significantly higher on smaller models. However, predation by birds did not differ between artificial models of varying signal luminance. This suggests that at the completion of metamorphosis, smaller froglets may be at a selective disadvantage, potentially because predators can discern they have relatively low levels of chemical defense compared to larger froglets. There is likely to be a premium on efficient foraging, giving rise to rapid growth and the acquisition of toxins from dietary sources in juvenile poison frogs.

## Introduction

Conditions during early stages of development are known to shape the later phenotype (Rossiter 1996; Monaghan 2008). In anurans, for example, these conditions may influence skin color (Ogilvy et al. 2012) and affect physiological condition (Jones et al. 2010; Crespi and Warne 2013), growth rate (LaFiandra and Babbitt 2004), and morphology (Touchon and Warkentin 2008). Aposematic species are distasteful or otherwise unprofitable and signal this property to predators with conspicuous coloration (Poulton 1890). Poison frogs are a group of aposematic animals that show high intraspecific variation in warning coloration (Lötters et al. 2007), but the consequences of signal development during immature stages for juvenile survival are unclear. In particular, during early development, resource allocation to growth and warning coloration can be constrained in aposematic species, as affected by the quantity or quality of nutrition (e.g. Grill and Moore 1998; Ojala et al. 2005; Blount et al. 2012). Indeed, it has been recently reported that availability of food during larval development in the aposematic green and black poison frog (*Dendrobates auratus*) affected body size and dorsal skin brightness (i.e., signal luminance), but not dorsal skin color (i.e., signal color or the main reflected wavelength) in postmetamorphic froglets (Flores et al. 2013). Aposematism exploits the innate and learned aversion of visually oriented predators toward conspicuous or novel colors, which results in increased predator wariness, enhanced avoidance learning rates, and thus reduced predation risk for the prey (Guilford 1986; Ruxton et al. 2004). Body size, color, and brightness contrast are key components of warning signals with the potential to influence predators' learning and avoidance (Ruxton et al. 2004; Stevens and Ruxton 2012). Furthermore, color and brightness contrast are known to facilitate detection, rejection and learning about warning signals in predators (Gamberale‐Stille 2001; Ham et al. 2006; Aronsson and Gamberale‐Stille 2013). Since predators differ in their visual sensitivity (Aidala et al. 2012; Moore et al. 2012), and how the cognitive and learning processes associated with visual stimuli take place (Kelber et al. 2003; Endler and Mappes 2004; Osorio and Vorobyev 2005; Stevens et al. 2009), any variation in the components of aposematic signals may be of critical importance for survival. For example, predators may attack novel aposematic prey more often (Noonan and Comeault 2009), monomorphism in warning signaling can result from anti‐apostatic selection (Allen and Greenwood 1988) or polymorphic signal design may be selected when the community of predators is variable (Endler and Mappes 2004). The propensity for some predators to attack prey despite the presence of warning coloration may impose a particular selective pressure on immature aposematic organisms, in which chemical defenses have not yet been developed or acquired, thus exposing them to high predation risk (see Gray and Christy 2000; Sime et al. 2000; Nylin et al. 2001). In particular, bird predators have been shown to taste‐reject aposematic prey based on their level of chemical defenses despite their similar warning appearance (Skelhorn and Rowe 2006).

Empirical evidence suggests that birds are important predators of aposematic species (e.g., Benson 1972; Exnerová et al. 2008) including poison frogs of the family Dendrobatidae (Cope 1865), in which warning colors appear to have evolved at least in part to confer protection against birds (Siddiqi et al. 2004; Maan and Cummings 2012). It has been previously reported that rufous motmots (*Baryphthengus martii*) prey upon poison frogs (Master 1999; Alvarado et al. 2013), while domestic hens (*Gallus domesticus*) have been shown to distinguish differences in conspicuousness and toxicity in poison frogs during predation experiments (Darst and Cummings 2006; Darst et al. 2006). Psychophysical models of bird vision have confirmed that birds can discern differences in terms of color and luminance (perceived level of brightness) of poison frogs (Maan and Cummings 2012), and in addition body size can affect aversion in birds (Forsman and Merilaita 1999; Jones and Osorio 2004). Nevertheless, the color, luminance and size of a signal may independently influence the perceptual psychology of birds and therefore affect rates of attack (Schuler and Roper 1992; Gamberale‐Stille and Tullberg 1999; Exnerová et al. 2010). Color is generally thought to primarily guide the detection and classification/discrimination of large objects and should be relatively constant under variable ambient light conditions (Osorio et al. 1999; Osorio and Vorobyev 2005). Luminance information is used in encoding object boundaries and texture, and detection of small targets and movement, and is more affected by changes in ambient light (Campenhausen and Kirschfeld 1998; Jones and Osorio 2004). There is some evidence that luminance contrast can also play a role in avoidance learning of aposematic prey in praying mantids (Prudic et al. 2007), and innate avoidance of undefended prey in the field by wild birds is enhanced by greater luminance contrast (Stevens et al. 2007). Therefore, color itself is likely to be important in learning of prey appearance and categorization of prey types, whereas luminance contrast and color contrast against the background may be important in initial detection and avoidance (Stevens and Ruxton 2012). Visual oriented predators in particular are known to avoid large body size and large pattern elements of warning signals (Gamberale and Tullberg 1998; Gamberale‐Stille 2000; Lindstedt et al. 2008). Indeed, larvae of some aposematic insects aggregate as a strategy to increase aversion in predators because in this way the signal size is enhanced (Gamberale and Tullberg 1998; Gamberale‐Stille 2000; Riipi et al. 2001).

Determining the consequences of specific aspects of aposematic signals for predation risk is difficult, because predator–prey interactions involving aposematic prey are rarely observed in the wild (though see Finkbeiner et al. 2012). Alternative experimental approaches that allow for the manipulation of aposematic phenotypes while at the same time measuring the responses of predators are more common. Artificial stimuli (models) made of plasticine or clay, for example, have been used to assess predation on aposematic amphibians (Saporito et al. 2007; Noonan and Comeault 2009; Chouteau and Angers 2011), reptiles (Brodie 1993; Wüster et al. 2004; Niskanen and Mappes 2005), and insects (Remmel and Tammaru 2009; Ihalainen and Lindstedt 2012). Observation of imprints left by predators (e.g., bites, beak marks) enables the identification of “predation” at different spatial and temporal scales. Nevertheless, it can be challenging to run experiments using artificial prey, for example, because of the need to correctly simulate prey coloration according to the visual sensitivities of putative predators. Visual systems are highly variable among taxa (Osorio and Vorobyev 2008), and thus, it is important to consider which predator(s) the experiment will target, considering the ecological and evolutionary context. While clay models can be deployed in the wild, they have been criticized due to their lack of movement (Cooper et al. 2009; Santos and Cannatella 2011). However, aposematic species in general rely on their warning signals for protection and exhibit slow motion (Ruxton et al. 2004).

Here, we present the results of a field study using clay models of *D. auratus* froglets deployed within their natural geographic distribution in Panama (see Köhler 2011) where the green and black morphotype is common. Our study builds on the results of a previous paper, Flores et al. (2013), in which froglets with access to relatively little food appeared to simultaneously maximize both body size and signal luminance, while froglets with access to greater amounts of food, which were larger on average, reduced their investment in signal luminance as compared to smaller individuals. Here, we address the relative importance of body size and signal luminance as determinants of survival in the wild. Thus, we prepared artificial models that varied in either body size (Experiment 1), or signal luminance as perceived by birds (Experiment 2), in order to test the effects of these two traits on rates of attack by bird predators. We hypothesized that variation in body size and signal luminance would influence the risk of predation. Specifically, if increased body size and signal luminance influence detectability and enhance the avoidance of predators, we predicted that (1) larger models would have higher survival than smaller models; and (2) individuals with greater signal luminance would have higher survival than those with lower signal luminance. Alternatively, if increased body size and signal luminance influence detectability but experienced predators are aware that recently metamorphosed froglets have little or no chemical defenses, the opposite predictions apply, namely, we predicted that (1) larger models would have relatively low survival; and (2) individuals with greater signal luminance would have relatively low survival.

## Materials and Methods

### Production of artificial stimuli

**Figure A1:**
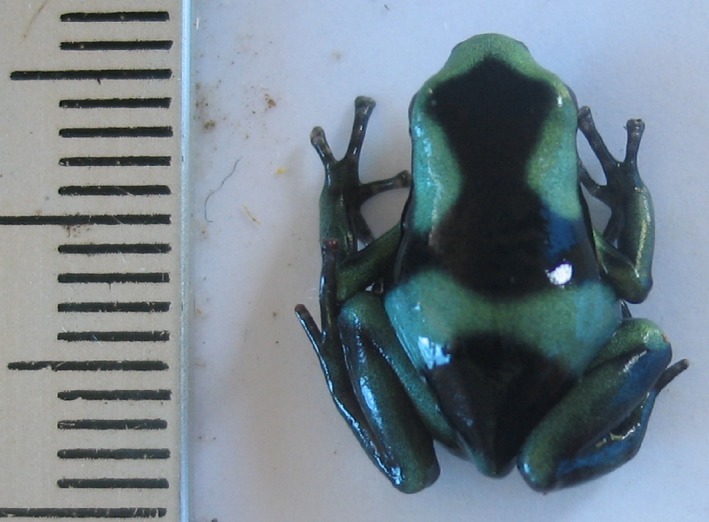
Dorsal view of the metamorphic juvenile used to design the contour and black dorsal pattern of artificial models. Each division in the scale represents 1 mm.

Artificial models were designed to resemble recently metamorphosed juveniles of *D. auratus*, which were themselves derived from a field‐based diet manipulation experiment carried out at Santa Fe, Veraguas province, during 2010 as described in Flores et al. (2013). Levels of body size (snout‐vent length; SVL) and luminance of artificial models were based on the results of the earlier diet manipulation experiment, in which dorsal luminance varied depending on SVL and food supply level. Body contour and design of the black dorsal pattern as seen from above were standardized, being measured using Image J 1.43q (Rasband 1997) from a digital image of the dorsum of one randomly chosen recently metamorphosed froglet collected at the field site. The image was taken with a Canon Power shot G6 (7.1 megapixel) digital camera (Canon Inc. Ohta‐ku, Tokyo, Japan) and later scaled to the experimental SVL values (Appendix Fig. A1). The proportion of the dorsum covered by black patterning was calculated using Image J 1.43q based on digital images of the dorsum of each experimental froglet in the high‐food and the low‐food supply groups, respectively, as described in Flores et al. (2013). The proportion of the dorsum covered in black patterning did not differ significantly between food groups (General Linear Mixed Model (GLMM); food: *F*_1,8_ = 3.27, *P *=* *0.11; mean ± SE = 0.58 ± 0.01%, *N *= 62). Moreover, the mean proportion of black pattern of experimental models did not significantly differ from a random sample of adults in the population (*F*_1,88_ = 2.96, *P *=* *0.089; froglets = 0.58 ± 0.01%, *N *=* *62; adults = 0.56 ± 0.01%, *N *=* *28). This proportion was therefore used for all artificial models. Dorsal signals are considered more important than ventral ones in warning signaling in dendrobatids (Wang and Shaffer 2008; Maan and Cummings 2012), and thus, we included only a black dorsal pattern in artificial models.

### Color and luminance discrimination

**Figure A2:**
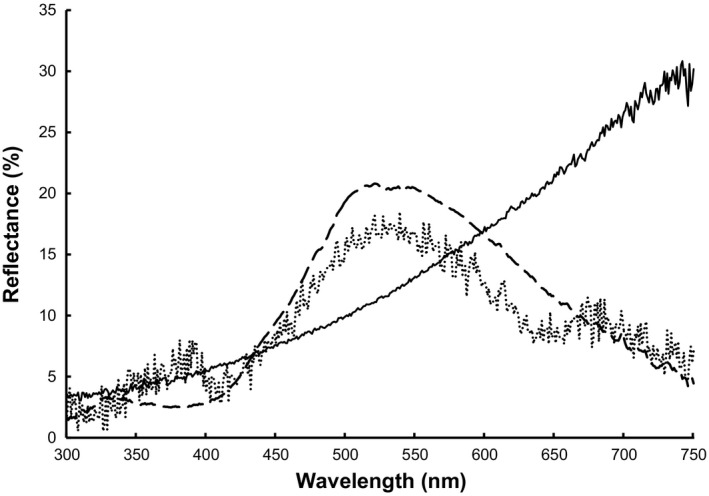
Mean reflectance spectra of: banana leaves used as natural substrate for the artificial models (solid line, *N *=* *12); randomly selected experimental froglets from Flores et al. (2013) (dashed line, *N *=* *10) and mixed clay of artificial models (dotted line, *N *=* *10).

**Figure A3:**
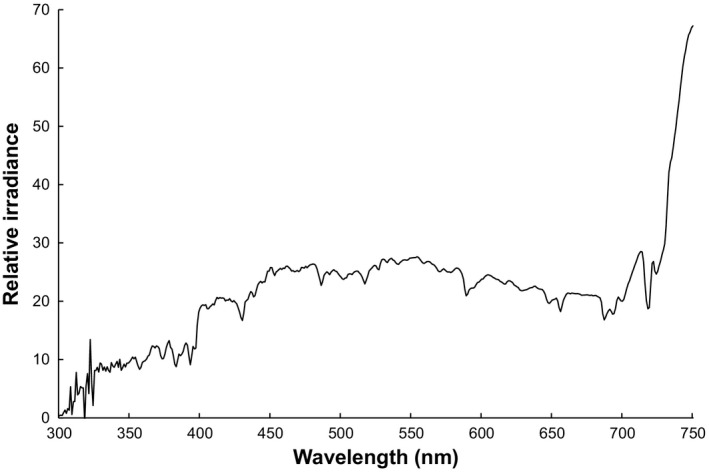
Mean irradiance spectra of ambient light at the study site. From Flores et al. (2013) with permission.

In birds, color and luminance discrimination are likely based on the sensitivity of single and double cone cell photoreceptors, respectively (Osorio and Vorobyev 2005, 2008). We used a variation of the Vorobyev–Osorio (V–O) visual model of color discrimination (Vorobyev and Osorio 1998), which has been employed to calculate discrimination values (i.e. just noticeable differences – JNDs) in intra‐ and interspecific studies of poison frogs (Siddiqi et al. 2004; Wang 2011; Maan and Cummings 2012). A JND value of 1 is considered as the threshold for discrimination, and values between 1 and 3 mean that two objects can probably only be discriminated under good viewing conditions (Siddiqi et al. 2004). To calculate photoreceptor sensitivity for the single (color sensitivity), double cones (luminance sensitivity), and the contrast of artificial prey signal against banana leaves as an ecologically realistic background, we first measured the spectral reflectance of clay with three replicates using a portable Jaz spectrometer (Ocean Optics Inc., Dunedin FL) with a bifurcated 400‐*μ*m UV/VIS fiber optic probe connected to an internal Jaz PX pulsed short arc xenon lamp (Ocean Optics Inc.). Measurements were made at an angle of 45° and corrected for lamp drift using a white diffuse spectral standard (WS‐1) (Maan and Cummings 2008). We measured the spectral reflectance of 12 dry banana leaves used as substrate for the artificial prey in triplicate and averaged them following the methodology described above (Appendix Fig. A2); subsequently, color and luminance discrimination were calculated between the banana leaves and the artificial models. We also measured ambient light irradiance at several locations in the field during 2010, *N *=* *90 measurements on a sunny day and *N *=* *85 measurements on a cloudy day, using a cosine corrected irradiance probe (CC‐3‐UV‐T) with 180° field of view connected to a USB2000 spectrometer (Ocean Optics Inc.) by means of a 400‐*μ*m UV/VIS fiber optic cable following the method described in (Endler 1993) (Appendix Fig. A3). The only known bird predator of *D. auratus*, the rufous motmot (*Baryphthengus martii*), is a near passerine (Livezey and Zusi 2007) and members of the family Momotidae have been reported to bear UV‐sensitive shortwave visual cones (Ödeen and Håstad 2013). As a proxy, we employed the blue tit (*Cyanistes caeruleus*) UV‐sensitive bird vision model, with tetrachromatic visual sensitivity (absorbance spectrum templates, oil droplets data, and relative number of receptor types from Hart et al. (2000) to simulate a potential bird predator vision system. Spectra were integrated over 1 nm intervals from 300 to 750 nm; details of calculations are provided in the Supporting Information. We used a *t*‐test to analyze contrast differences between the black and green regions on the artificial prey in the two luminance groups in this experiment. We found significant contrast differences between the black painted spots and the green colors of the artificial models in the two luminance treatments (*t*‐test; *t*_5.681_ = 32.10, *P *<* *0.001, High luminance = 30.84 JND, Low luminance = 26.52 JND).

### Experiment 1, effect of body size variation

Five prey phenotypes (S1–S5) were designed to be equally spaced in increments of size (i.e. 0.846 mm) along the distribution of SVL values (Table 1). As we were only interested in the effect of body size, we held constant the values of color contrast sensitivity and luminance contrast sensitivity, according to the average of both experimental high‐ and low‐food supply froglets. To prepare the artificial prey, nontoxic, Sculpey III^®^ clay (Polyform Products Co., Elk Grove Village, IL) and Fimo soft^®^ clay (Staedtler Mars, GmbH & Co. Nürnberg, Germany) were manually mixed. Details of clay mixing are provided in the Supporting Information.

**Table 1:** Artificial model phenotypes in terms of snout‐vent length (SVL) used for Experiment 1

Artificial model phenotype (SVL, mm)				
S1	S2	S3	S4	S5
14.45	15.30	16.14	16.99	17.84

### Experiment 2, effect of signal luminance

**Figure A4:**
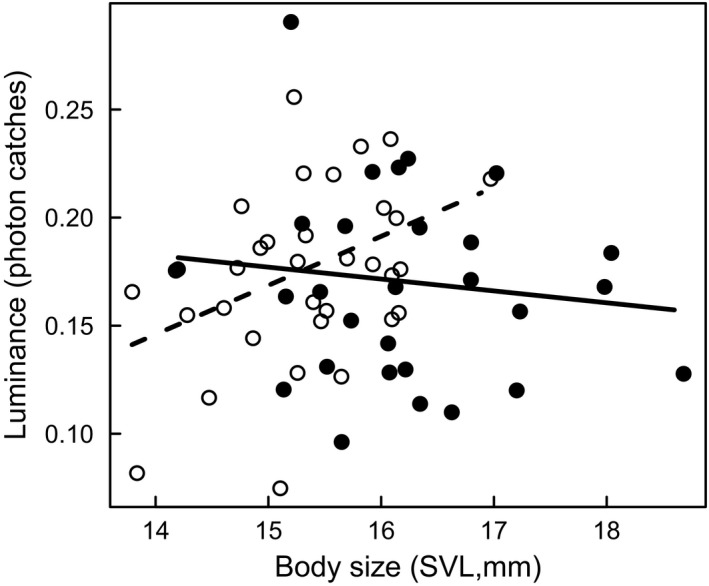
Relationship between dorsal luminance (modeled based on bird vision) and snout‐vent length (SVL) in froglets of the two food supply groups. Filled circles and solid line: high‐food individuals; open circles and the dashed line: low‐food individuals. From Flores et al. (2013) with permission.

In our design, the artificial model phenotype “S2” represents the body size as indicated in Flores et al. (2013), after which high‐food supply froglets exhibited reduced signal luminance (Appendix Fig. A4). Therefore, to determine the effect of luminance variation, the median values of SVL in the upper (75–100%) interquartile range for the high‐ and low‐food supply froglets were calculated and averaged to obtain a single large body size (i.e. 16.7 mm) in the distribution of SVL. This size was then used to obtain the corresponding luminance values using equations following results in (Flores et al. 2013), see Supporting Information for details. These calculations generated a High level = 0.21 and a Low level = 0.17 of luminance, enabling us to test the effect of signal luminance on predation risk in large postmetamorphic individuals.

### Digital design of artificial models and mold preparation

Artificial models were digitally designed using SolidWorks 3D CAD 2011 SP 4.0 software (Dassault Systèmes SolidWorks Corp., Waltham, MA), simulating a *D. auratus* individual in a natural sitting posture. Details of the manufacturing process are given in the Supporting Information. In order to deploy the models, they were glued to the blade of a standard shaped 15 × 10 cm piece of dry banana leaf, which is a typical substrate at our study site, using a small dab of Loctite Epoxi‐mil epoxy adhesive (Henkel corporation, Düsseldorf, Germany).

### Similarity between artificial models and froglets

**Figure A5:**
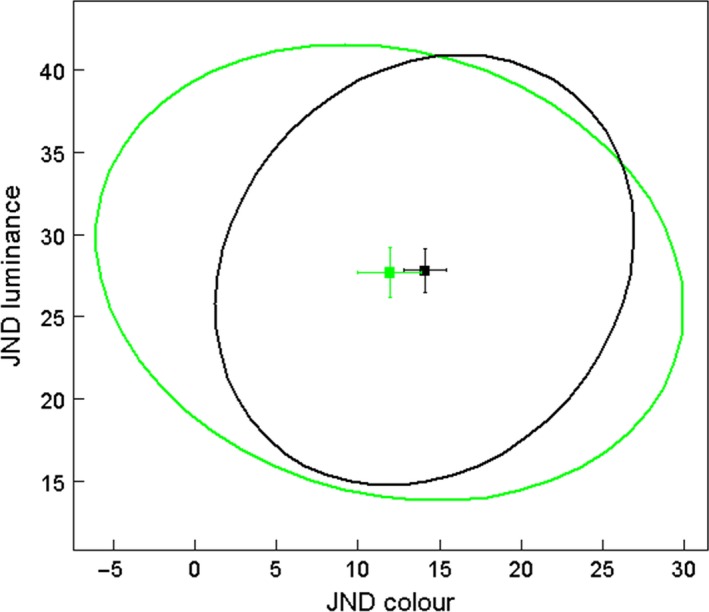
Comparison of overall conspicuousness of the black pattern painted on artificial models (*N *=* *12) and those of randomly selected froglets (*N *=* *10) against a banana leaf substrate as viewed by a bird predator. The *x*‐axis is JND color contrast, and the *y*‐axis JND luminance contrast. Ellipses show 95% confidence interval for artificial models (black line) and experimental froglets (green line). Black and green squares are the mean ± SE for artificial models and experimental froglets from Flores et al. (2013).

**Figure A6:**
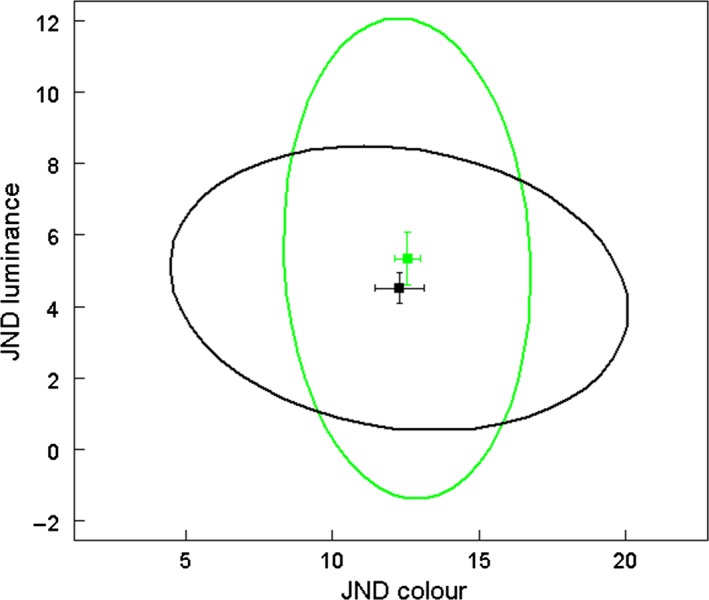
Comparison of overall conspicuousness of the mixed clay of artificial models (Experiment 1) (*N *=* *10) and those of randomly selected froglets (*N *=* *10) against the banana leaves substrate as viewed by a bird predator. The *x*‐axis is JND color contrast, and the *y*‐axis JND luminance contrast. Ellipses show 95% confidence interval for mixed clay (black line) and experimental froglets (green line). Black and green squares are the mean ± SE for mixed clay of artificial models (Experiment 1) and experimental froglets from Flores et al. (2013).

JND luminance and color contrast did not differ significantly between the black pattern painted on artificial models (*N *=* *12) and the natural black pattern of randomly selected froglets (*N *=* *10) derived from the experiment described in Flores et al. (2013) (JND luminance: GLM, *F*_1,20_ = 0.01, *P *=* *0.94; log(JND color): GLM, *F*_1,20_ = 1.71, *P *=* *0.20; Appendix Fig. A5). Similarly, JND luminance did not differ significantly between mixed clay and the same experimental froglets (JND clay ± SE = 4.51 ± 0.60, *N *=* *10; JND frog ± SE = 5.34 ± 0.85, *N *=* *10; JND luminance: GLM, *F*_1,18_ = 0.51, *P *=* *0.48). A qualitatively similar result was found for JND color contrast (JND clay ± SE = 12.28 ± 0.68, *N *=* *10; JND frog ± SE = 12.55 ± 0.96, *N *=* *10; log(JND color): GLM, *F*_1,18_ = 0.24, *P *=* *0.63) (Appendix Fig. A6). Dorsal skin in dendrobatids mostly lacks UV reflectance (Summers et al. 2003; Noonan and Comeault 2009), and similarly, experimental froglets did not show appreciable levels of UV reflectance in their dorsal skin (Flores et al. 2013). Accordingly, we found that the UV reflectance of our mixed clay was low (UV mixed clay ± SE: 0.077 ± 0.002, *N *=* *10); therefore, it was unlikely to influence our results. JND for color was not significantly different among artificial models (*F*_1,6_ = 5.55, *P *=* *0.06; Table 2). However, JND for luminance was significantly different among artificial models (*F*_1,6_ = 685.8, *P *<* *0.001; Table 2). In general, all JND values of artificial prey were higher than three; indicating that our modeled bird predator could discriminate between models and the banana leaf background.

**Table 2:** JNDs of artificial models from Experiment 1 (effect of body size) and Experiment 2 (effect of signal luminance) against banana leaf background. JNDs were calculated as the discrimination between two spectral stimuli following the V–O model (see Supporting information for details of vision model). Values are mean ± SE

	*N*	JND luminance	JND color
Experiment 1			
	10	4.51 ± 0.60	12.28 ± 0.68
Experiment 2			
LL	5	3.49 ± 0.10	8.50 ± 0.17
LH	3	7.81 ± 0.16	7.84 ± 0.28

### Deployment of models

Artificial models were deployed in the field during the rainy season of 2011 at the end of May for Experiment 1 and at the beginning of August for Experiment 2, at a shade organic coffee plantation in Santa Fe, Veraguas province, central Panama (8°31′ N 81°03′W). For Experiment 1, we deployed a total of *N *=* *600 models, and for Experiment 2, a total of *N *=* *240 models. We used a randomized block design, in which each block (*N *=* *6), contained either *N *=* *100 models (20 of each phenotype for Experiment 1) or *N *=* *40 models (20 of each phenotype for Experiment 2), deployed randomly along nonlinear zig‐zag transects, maintaining an approximate minimal distance of 10 m among models and 50 m among blocks (Cuthill et al. 2005; Rowland et al. 2008; Stevens et al. 2008). As *D. auratus* performs a daytime foraging behavior on the surface of leaves, tree trunks, or logs (Toft 1981; Savage 2002), all models were deployed on a piece of dry banana leaf as a common and natural substrate in a typical sitting posture exposing their dorsal area. Blocks were deployed one at a time, with all the models in a single block deployed the same day early in the morning. Monitoring of models was performed on a daily basis 24 h after deployment following the same order and for a total of seven days per block. Experiment 2 started at the same study site two weeks after Experiment 1 had concluded, in order to minimize any possible effects of learning by our target predators.

### Statistical analyses

Analyses were conducted using R v.2.12.1 (R Development Core Team, 2010). Survival analysis was performed using Cox proportional‐hazards regression (Cox 1972). This nonparametric survival analysis allows inclusion of censored records (i.e. nonavian predation) providing more information to the survival function (Cuthill et al. 2005). Models with U‐ or V‐shaped beak marks (Brodie 1993; Hegna et al. 2011) were classified as attacked by birds and were therefore removed, photographed, and recorded as dead. Models attacked by mammals (clear marks of incisor teeth), with unidentified marks, complete disappearances and those which were not attacked were recorded as censored. The proportional criteria of the Cox model were tested based on the GLOBAL test, with a resulting *P *=* *0.337 indicating our data met the criteria. We also tested for the effect of block per se; its inclusion as a random factor did not qualitatively change the results, and therefore, we present results for models that do not include block as a random factor. In Experiment 1, when there was a significant effect of model size on survival, planned comparisons based on the Wald statistic between pairs of models were conducted and the hazard ratio with corresponding confidence intervals between pairs also reported. In Experiment 2, the effect of luminance on large models was also tested using the Wald test. Here, the hazard ratio represents the multiplicative average effect of one category of model with respect to the other on the hazard related to the incidence of being killed or risk of mortality. To test whether the probability of attack by birds differed between Experiments 1 and 2, we conducted a binomial logistic regression including the estimates of effects (i.e. odds ratio) (see Hegna et al. 2011). Here, the odds ratio represents the ratio of the odds of attack in Experiment 1 to the odds in Experiment 2. *P *<* *0.05 was considered statistically significant in all analyses.

## Results

### Experiment 1: effect of body size on predation risk

**Figure 1:**
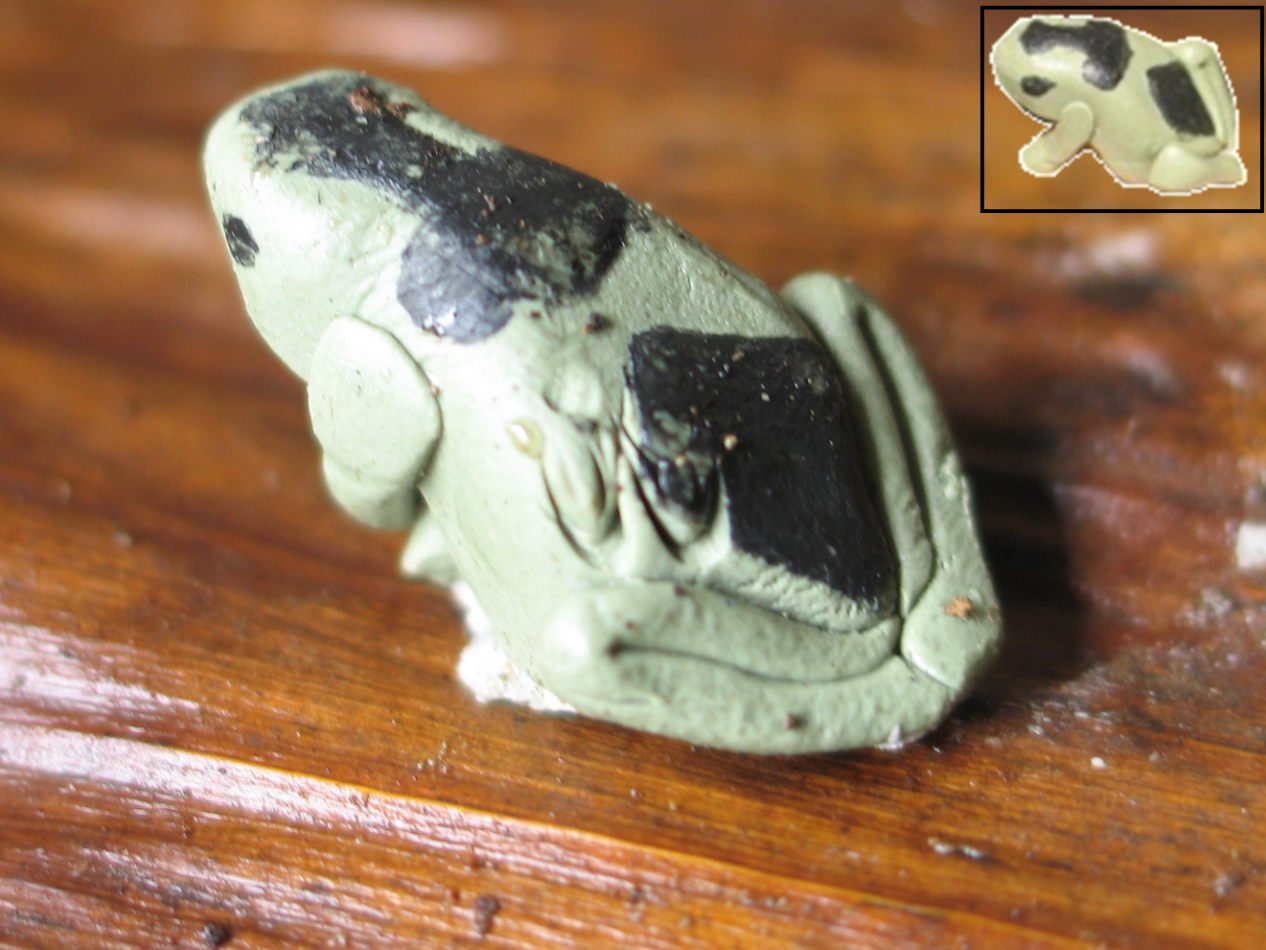
Juvenile *Dendrobates auratus* artificial model on banana leaf substrate, with beak mark imprints. Inset showing an original intact model.

**Figure 2:**
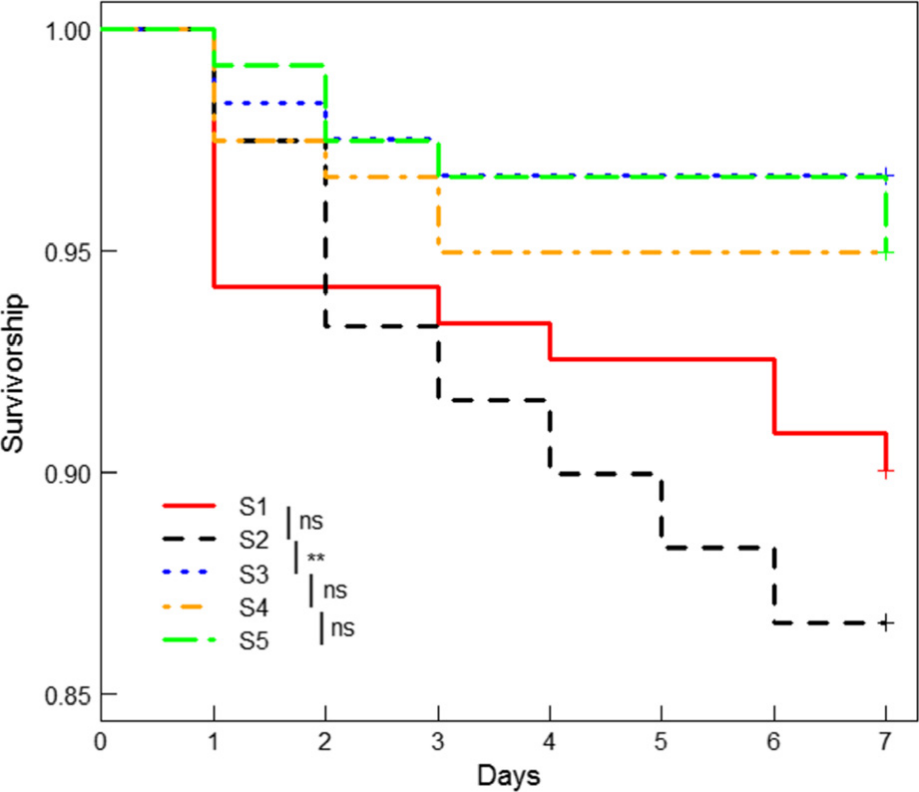
Cumulative survivorship curves for five categories of body size of artificial models over 7 days. See Table 1 for details of size categories. Smaller artificial models (S2) survived significantly less compared with larger models. Vertical bars in the legend represent the planned comparisons conducted between pairs of artificial model categories; ns: not significant.

A total of 44 of 597 artificial prey were attacked by birds (7%) (Figs. 1 and 2), whereas 34 prey were attacked by unknown predators (6%), while three models could not be re‐found and were classed as censored. Overall smaller prey survived less than larger prey (Fig. 2; Cox regression; *χ* ^2^_4_ = 11.84, *P *=* *0.02). This conclusion was unchanged by the inclusion of block as a random factor. Survival of the smallest prey was not significantly different from the threshold sized prey (S1 vs. S2; hazard ratio = 1.35, CI_95%_ = 0.64–2.86, Wald *χ* ^2^_1_ = 0.63, *P *=* *0.43), although the S2 prey survived significantly less well compared with the next size category (S2 vs. S3; hazard ratio = 0.24, CI_95%_ = 0.08–0.71, Wald *χ* ^2^_1_ = 6.57, *P *=* *0.01). Survival of prey in category S3 was not significantly different from category S4 (hazard ratio = 1.52, CI_95%_ = 0.43–5.40, Wald *χ* ^2^_1_ = 0.43, *P *=* *0.51), and a similar result was found for categories S4 vs. S5 (hazard ratio = 0.99, CI_95%_ = 0.32–3.07, Wald χ^2^_1_ = 0, *P *=* *0.98). Survival of models attacked by unknown predators occurred independently of size (χ^2^_4_ = 6.60, *P *=* *0.16).

### Experiment 2: effect of signal luminance on predation risk

There were a total of eight of 235 models attacked by birds (3%), and 21 models were attacked by unknown predators (9%), while five models could not be re‐found and were classed as censored. Signal luminance was not a significant predictor of survival in larger artificial prey (High luminance vs. Low luminance; hazard ratio = 3.04, CI_95%_ = 0.61–15.06; Wald *χ* ^2^_1_ = 1.85, *P *=* *0.17). This conclusion was unchanged by the inclusion of block as a random factor. Similar results were found when attacks by unknown predators were considered (*χ* ^2^_1_ = 0.03, *P *=* *0.87).

The probability of attacks in Experiment 2 was half that recorded in Experiment 1 (odds ratio = 2.16, CI_95%_ = 1.10–4.15; Wald *χ* ^2^_1_ = 5.07, *P *=* *0.024).

## Discussion

This study aimed to evaluate the consequences of early environmental conditions for predation risk in an aposematic frog species. Use of artificial models has proven to be a useful technique for understanding how predators respond to variation in warning signals (Benson 1972; Lindström 1999; Chouteau and Angers 2011). Several previous studies have taken into account the visual system of the potential predator in the design of artificial prey (Stevens et al. 2007, 2008; Rowland et al. 2008), although to our knowledge the present study is among the first to have used this approach in poison frogs (but see Stuart et al. 2012).We found that larger body size in artificial models resulted in reduced predation risk by birds compared with smaller models. Our study therefore suggests that lower attack rates by birds on larger postmetamorphic *D. auratus* could contribute to the selective pressures favoring large size. Contrary to our predictions, predation by birds did not differ between artificial models that varied in terms of signal luminance; this could result from relaxed selection on this aposematic trait during early life stages or lack of statistical power in our design.

### Effect of body size

We found that birds avoided attacking larger artificial models. This is contrary to the prediction that larger postmetamorphic *D. auratus* would suffer greater predation because of increased detectability. Body size has been shown to be a predictor of detectability in early larval stages of the caterpillar *Orgyia antiqua* (Sandre et al. 2007). However, attack rates by bird predators have been found to be negatively correlated with body size in artificial prey of this species (Mänd et al. 2007). This could be related to the increased effect of the warning signal in larger prey (Remmel and Tammaru 2011). Birds may not necessarily learn about differences in prey defenses based on body size alone and rather make use of warning signaling (Halpin et al. 2013). One possible explanation for our results, therefore, is that larger artificial models were more aversive to bird predators because predators have an innate wariness of large warning signals (Gamberale and Tullberg 1996, 1998; Forsman and Merilaita 1999) or have learned that larger froglets tend to have greater defensive capacity (Hagman and Forsman 2003; Santos and Cannatella 2011). It is also possible that larger models may have benefitted from reduced attack rates by birds in part because they had greater resemblance to adults (i.e. automimicry, Speed et al. 2006). However, we note that even the largest of our experimental models (i.e. 17.84 mm) was considerably smaller than the size normally attained by adult *D. auratus* in the wild (i.e. 40 mm) (Köhler 2011).

Interestingly, artificial models in the two smallest size categories (S1 = 14.45 mm; S2 = 15.30 mm) had relatively low survival, compared with all size classes of larger artificial models (see Fig. 2). This result supports the idea of a perceptual size threshold beyond which survival increases or is maintained without further beneficial effects of increments in body size (Forsman and Herrström 2004). Notably, the two smallest size categories in our experiment were similar to the SVL reported for recent metamorphic *D. auratus* froglets in the wild (range: 14.0–14.8 mm; Eaton 1941; Pope 1941). Body size in anurans is also linked to survival (Morey and Reznick 2001), may influence dispersal (Pough and Kamel 1984), foraging ability (McCallum and McCallum 2012), and mating success (Arak 1988), and has been reported to correlate positively with the strength of warning signals (Hagman and Forsman 2003; Santos and Cannatella 2011), suggesting an association between these phenotypic traits as one mechanism for the evolution of aposematism. This association has been strongly linked to diet specialization in terms of the acquisition of alkaloid‐bearing arthropods (Santos and Cannatella 2011). Consequently, we may expect small juveniles in the population to be more vulnerable than those with larger body size, due to a lower capacity to acquire and store secondary defenses (Daly et al. 2002; Saporito et al. 2010). Indeed, dietary sequestration of alkaloids begins just after metamorphosis in *D. auratus* (Daly et al. 1994; Saporito et al. 2009) which might mean they are particularly vulnerable to predators as young adults. As birds are capable of differentiating prey of different sizes (Gamberale and Tullberg 1996; Grieco 2002), and also seem to detect differences in alkaloid defense levels in poison frogs (Darst and Cummings 2006; Darst et al. 2006), it could be that birds at our study site selectively attack froglets that are smaller than a certain threshold, and therefore similar in body size to recent metamorphic, poorly defended froglets. Evidence suggests that birds cannot readily distinguish among relatively small differences in sizes of defended prey, until they have gained experience with a larger size difference (Marples 1993). Our results suggest that bird predators may have been experienced and employed a capability to distinguish sizes, being able to differentiate palatable from unpalatable *D. auratus* froglets based on rather small differences in body size. It is known that both pattern element size and body size of prey enhance the effectiveness of warning signals. However, in our experiments with artificial models, the black pattern area varied in proportion with body size and therefore we cannot separate the influence of these traits on prey survival.

### Effect of luminance

Although luminance contrast can be an effective warning signal alone (Prudic et al. 2007), our results show that luminance variation did not significantly explain differences in attack rates of artificial models. As demonstrated previously, conspicuous signaling does not necessarily reduce attack rates in small prey (Niskanen and Mappes 2005; Mänd et al. 2007). It could be that lack of mobility of the artificial prey impaired the perception of luminance by bird predators; however, levels of JND luminance of the two artificial prey phenotypes in Experiment 2 against a banana leaf background were discriminable to the modeled bird vision system (i.e. both >3.0) (see Table 2). One possibility is that the relatively small luminance differences among artificial models did not reach the threshold at which birds can discern and respond in terms of different attack rates. This will require further experimentation.

Artificial prey constructed from clay obviously lack mobility, which reduces the realism of this methodology. Although not testing for luminance variation per se, Paluh et al. (2014) found that aposematic color was a predictor of predation rates by birds of continuing moving models, but not stationary models. Predators are more likely to attack moving prey (Heinen and Hammond 1997), but nevertheless, continuously moving models may not accurately represent the behavior of aposematic species. Typically, aposematic prey exhibit slow motion, reduced escape distance, and move slowly near predators (Ruxton et al. 2004; Cooper et al. 2009). Luminance perception can be strongly affected by environmental light conditions (Osorio and Vorobyev 2005), especially in the tropical forest understory where gaps of light and shadows are common (Théry 2001). Therefore, the complex background environment of the forest floor may have rendered birds unable to discern differences in luminance, or at least it was not a reliable cue to be used in discrimination. It should also be noted that in complex habitats other factors can interact to influence the perception of prey, for example distance, shadows, and countershading (Tullberg et al. 2005; Rowland et al. 2008); this requires further study.

Another possibility is that selection imposed by birds on signal luminance is weak at our study site. Although birds seem to show innate wariness toward conspicuous colors that are generally associated with aposematic species (Schuler and Roper 1992; Lindström et al. 1999; Exnerová et al. 2007), empirical studies have demonstrated that contrasting colors in aposematic prey do not affect rates of predation by birds in the wild (Noonan and Comeault 2009; Chouteau and Angers 2011; Hegna et al. 2013), although these studies did not specifically test for variation in luminance contrast while the color of the signal was kept constant. Arguably, the green and black markings of *D. auratus* and our artificial prey may be considered weak warning colors (Stevens and Ruxton 2012). However, there is extreme variation in the proportion of these two colors among different populations of *D. auratus* (Lötters et al. 2007), which could markedly affect recognition errors by predators, especially in the forest. Thus, weak or moderately conspicuous signals may be selected for because they reduce detection, especially if a fraction of predators manage to overcome the defenses of prey individuals or are naïve (Endler and Mappes 2004; Speed and Ruxton 2007). The internal luminance contrast between the black and green colors of our artificial prey in Experiment 2 differed between the two luminance groups, and in both cases was well above the minimal threshold value for discrimination (i.e. JND = 1). Thus, following detection, differences in conspicuousness within the body of the prey could in theory have influenced attack decisions. It would be interesting to test whether predation risk is affected by different levels of internal luminance contrast, including variation in “typical” aposematic colors (i.e. red, orange, yellow). Finally, it is notable that numbers of attacks on artificial models in the luminance variation experiment were only half that observed in the size variation experiment. This could be because models in the luminance experiment were all relatively large (and larger prey are less likely to be attacked). However, a lower predation rate does of course mean‐reduced statistical power to detect any effect of luminance variation, even if it had existed.

Size‐dependent predation risk may impose selection pressures on antipredator strategies employed during early life stages in aposematic species. For example, it could be beneficial to remain small if size correlates positively with detectability (Higginson and Ruxton 2009), in particular where predators are naïve with respect to prey defenses. In contrast, we found that the smallest artificial prey had the lowest survival. *Dendrobates auratus* froglets must face a particularly high risk of predation in the critical days and weeks following metamorphosis, when they must forage to acquire and accumulate toxins while also growing to attain adult body size. Indeed, it seems likely that individuals which are larger at metamorphosis will subsequently acquire toxins more quickly, because larger individuals may have a higher aerobic capacity and hence greater foraging efficiency (Santos and Cannatella 2011). Nevertheless, conspicuous appearance alone is insufficient to confer complete protection against predators (Endler and Mappes 2004; Mappes et al. 2005); larger, more conspicuous juveniles may face increased inspection and “handling” by predators (Mänd et al. 2007). It would therefore be interesting to observe how investment in aposematic signaling may change as individuals acquire toxins postmetamorphosis. Individuals could benefit by reducing signal conspicuousness as their body size and levels of chemical defense increase. Less conspicuous but more toxic juveniles would likely have reduced encounter rates with different predators, but in the event of an attack they are more likely to survive (Leimar et al. 1986; Speed and Ruxton 2007).

Our experiment targeted a specific bird vision system predator, however other animals have also been reported as predators of poison frogs, including the Red Rump Tarantula (*Sericopelma rubronitens*) (Summers 1999), and the Macabi Tetra (*Brycon guatemalensis*) (Hedstrom and Bolaños 1986). At our study site, it is common to observe birds including Blue‐crowned Motmot (*Momotus momota*), Gray‐necked Wood Rail (*Aramides cajanea*), Pale‐vented pigeon (*Columba cayennensis*), Smooth‐billed Ani (*Crotophaga ani*) and domestic hens (*Gallus domesticus*). In addition, reptiles that occur at our study site include Green Tree Anole (*Norops biporcatus*), Central American Coral Snake (*Micrurus nigrocinctus*), Fer‐de‐lance (*Bothrops asper*), Neotropical Bird Snake (*Pseustes poecilonotus*), and Red Coffee Snake (*Ninia sebae*). Which (if any) of these species were responsible for attacks on artificial models is not known. While we lack a detailed synthesis of the range of taxa that attack *D. auratus* in the wild, our results at least for artificial models suggest that the range of predators may include nonavian taxa.

In conclusion, our study of artificial models suggests that early environmental conditions affecting body size in postmetamorphic aposematic froglets may have an important influence on rates of attack by bird predators. This could potentially be because bird predators can discern the relationship between body size and likely defensive capacity derived from dietary sources. Whether this association between body size and predation risk also applies in fully grown adult prey merits further research. Overall, our results based on predation risk imposed by birds add to the group of selective pressures imposed on body size in early postmetamorphic *D. auratus*.

## Conflict of Interest

None declared.

## Supporting information

Data S1. Further methodological details.
