# Supplementary material for: Body size but not warning signal luminance influences predation risk in recently metamorphosed poison frogs
Source: Ecol Evol. 2015 Oct 5;5(20):4603–16. doi: 10.1002/ece3.1731 (PMC4670055; doi:10.1002/ece3.1731)
Supplement: Supplementary file 1 — Data S1. Further methodological details. [file ECE3-5-4603-s001.docx]

**Modeling predator vision and artificial prey conspicuousness**

Calculations were done based on a set of functions in Matlab R2009a (The MathWorks Inc, USA). In birds color perception stems from the comparison of the relative stimulation of the different single cones sensitive to ultraviolet (UV), short (SW), medium (MW) and long (LW) wavelengths with opponent color channels (Kelber, Vorobyev, & Osorio, 2003). Meanwhile luminance sensitivity (achromatic) appears to be based on the stimulation of the double cone photoreceptors (Kelber et al., 2003; Osorio & Vorobyev, 2005). We used the blue tit (*Cyanistes caeruleus*), which has an ultraviolet shifted ultrashortwave cone type, as a tetrachromatic visual model to calculate predicted photon catches for the different cone types. For luminance, we used an extension of the model using double cones (Siddiqi, Cronin, Loew, Vorobyev, & Summers, 2004). The model starts by calculating the cone quantum catches (*qi*) for each photoreceptor class for froglets or clay and ambient radiance spectra as:

$${qi}_{bird}= \int_{\lambda=300}^{750} R(\lambda)I\left( \lambda\right)Aᵢ\left( \lambda\right)d\lambda,$$

Equation S1. Calculation of cone quantum catches of photoreceptors of the bird vision model.

here $A_{i}\left( \lambda\right)$ represents the absorptance spectrum for each of the four photoreceptor cone classes of the bird integrated over 1 nm intervals from 300 to 750 nm $R\left( \lambda\right)$and $I\left( \lambda\right)$ represent the reflectance spectrum of the froglet´s skin or clay and the irradiance spectra measured in the field respectively. Resulting photon quantum catches were standardized in order to account for variation in light conditions, using the von Kries transformation adaptation coefficient. This method assumes that photoreceptors adjust their sensitivity in proportion to the background light environment:


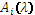

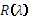

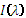

$${Ki}_{bird}= \left( \int_{\lambda=300}^{750} Ai\left( \lambda\right)I\left( \lambda\right)d\lambda\right)^{-1},$$

Equation S2. von Kries transformation adaptation coefficient of photoreceptors of the bird vision model.

This procedure ensures that color perception relies on color constancy, whereby the visual system removes variation in ambient light so that colors look similar under variable light conditions (Cuthill, 2006). Therefore, the adjusted quantum catch data for each photoreceptor class was calculated as:

$$q_{a}={Ki}_{bird} x {qi}_{bird},$$

Equation S3. Adjusted quantum catches of each photoreceptor of the bird vision model.

Following calculations of quantum catches, the model assumes that the signal of each cone channel is proportional to the logarithm of the adjusted quantum catches; as such the contrast between a pair of stimuli was calculated as the quotient of adjusted photon quantum catches:

$$\Delta q_{a}=log\frac{\left[ q_{a1}(frog spectra) \right]}{\left[ q_{a2}(background spectra) \right]}$$

Equation S4. Calculations of contrast between a pair of stimuli of each photoreceptor of the bird vision model.

The Vorobyev-Osorio color discrimination model is based on evidence that color discrimination is determined by noise arising in the photoreceptors and is independent of light intensity. Noise in each photoreceptor channel ${(e}_{i})$ was calculated as:


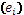

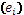

$$e_{i}= \frac{\omega_{i}}{\sqrt{n_{i}}}$$

Equation S5. Calculation of noise in each photoreceptor of the bird vision model.

where $\omega_{i}$ was taken as 0.05 and represents the Weber fraction of the most abundant cone type (Siddiqi et al., 2004) and $n_{i}$ is the relative number of receptor types in the retina of the blue tit (Hart, Partridge, Cuthill, & Bennett, 2000) $\left( n_{L}= 1.00, n_{M}= 0.99, n_{S}=0.71, n_{UV}=0.37 \right)$. Color (chromatic) discrimination in the tetrachromatic visual model was calculated as JND values using the following equation:


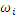

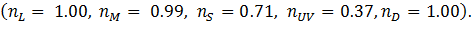

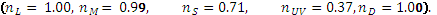

$$JND bird color=\sqrt[2]{\frac{\begin{aligned} \left( e_{UV}e_{S} \right)^{2}\left( {\Delta q}_{L}-\Delta q_{M} \right)^{2}+\left( e_{UV}e_{M} \right)^{2}\left( {\Delta q}_{L}-{\Delta q}_{S} \right)^{2}+\left( e_{UV}e_{L} \right)^{2}\left( {\Delta q}_{M}-{\Delta q}_{S} \right)^{2} \\ +\left( e_{S}e_{M} \right)^{2}\left( {\Delta q}_{L}-{\Delta q}_{UV} \right)^{2}+\left( e_{S}e_{L} \right)^{2}\left( {\Delta q}_{M}-{\Delta q}_{UV} \right)^{2}+\left( e_{M}e_{L} \right)^{2}\left( {\Delta q}_{S}-{\Delta q}_{UV} \right)^{2} \end{aligned}}{\left( e_{UV}e_{S}e_{M} \right)^{2}+\left( e_{UV}e_{S}e_{L} \right)^{2}+\left( e_{UV}e_{M}e_{L} \right)^{2}+\left( e_{S}e_{M}e_{L} \right)^{2}}}$$

Equation S6. Just noticeable differences (JND´s) for color discrimination of the bird vision model.

Since in our model, overall perceived luminance is considered to arise from stimulation of double cone photoreceptors, luminance discrimination was evaluated as:

$$JND bird luminance=\left( \frac{{\Delta q}_{D}}{e_{D}} \right)$$

Equation S7. Just noticeable differences (JND´s) for luminance discrimination of the bird vision model.

Color and luminance discrimination (conspicuousness) was based on JND values.

**Details of clay mixing for Experiment 1**

Clay was weighed to the nearest 0.001g using an Ohaus Scout Pro balance (Ohaus Europe GmbH, Switzerland). Sculpey clay types were: 001 White, 042 Black and 1629 Granny Smith. Fimo clay types were: 26 Cherry Red and 37 Blue. To prepare 100 g of the average color and luminance for Experiment 1, clay was mixed as follows: 63g of 1629 Granny Smith, 19g of 042 Black, 7g of 26 Cherry Red, 3.5g of 001 White, 3.5g of 37 Blue, 2.5g of 001 White and 2.5g of 042 Black.

**Equations used to calculate Luminance values for Experiment 2**

$${Luminance}_{high food} (photon catches)=0.26-0.005*SVL$$

Equation S8. Modeled equation used to predict the luminance values based on snout-vent length (SVL) in froglets of the high-food treatment.

$${Luminance}_{low food} (photon catches)= -0.17+0.023*SVL$$

Equation S9. Modeled equation used to predict the luminance values based on snout-vent length (SVL) in froglets of the low-food treatment.

The two values obtained represent luminance photon catches of real froglets, and span 4 JNDs in terms of contrast against a banana leaf background. To obtain these values using the modeling clay, we added to 100 g of the average color mixture of Experiment 1 the following: 4 g of 001 White to obtain value High luminance, and 2g of 001 White to obtain value Low luminance.

**Digital design of artificial models and mold preparation**

Digital files were exported in STL format ready for the manufacturing process and transferred to a Roland MDX 500 automatic milling machine (Roland DGA Corp., California, USA) by means of Mayka Expert 7.0 software (PicaSoft, France). Molds for each specific size class were drilled in two steps to increase precision using a 3.0 mm ball nose on a 15.0 x 15.0 x 2.5 cm (L x W x H) block of resin board. Individual artificial prey were made by pressing the experimental mixed clay into the specific mold, carefully extruding the clay, and finishing by removing any excess of clay with a scalpel. The black dorsal pattern and eyes were manually applied using non-toxic black poster paint (Sargent Art Inc, Hazleton PA, USA). Front and hind legs were manually attached using the appropriate mixed clay for Experiment 1 and Experiment 2, respectively.

Literature cited

Cuthill, I. C. (2006). Color perception. In G. E. Hill & K. J. McGraw (Eds.), *Bird coloration, vol. I: Mechanisms and measurements* (pp. 3–40). Cambridge: Harvard University Press.

Hart, N. S., Partridge, J. C., Cuthill, I. C., & Bennett, A. T. D. (2000). Visual pigments, oil droplets, ocular media and cone photoreceptor distribution in two species of passerine bird: the blue tit (Parus caeruleus L.) and the blackbird (Turdus merula L.). *Journal of Comparative Physiology A*, *186*(4), 375–387. doi:10.1007/s003590050437

Kelber, A., Vorobyev, M., & Osorio, D. (2003). Animal colour vision – behavioural tests and physiological concepts. *Biological Reviews*, *78*, 81–118.

Osorio, D., & Vorobyev, M. (2005). Photoreceptor sectral sensitivities in terrestrial animals: adaptations for luminance and colour vision. *Proceedings of The Royal Society of London B*, *272*, 1745–1752. doi:10.1098/rspb.2005.3156

Siddiqi, A., Cronin, T. W., Loew, E. R., Vorobyev, M., & Summers, K. (2004). Interspecific and intraspecific views of color signals in the strawberry poison frog Dendrobates pumilio. *Journal of Experimental Biology*, *207*, 2471–2485.
